# The DUBm subunit Sgf11 is required for mRNA export and interacts with Cbp80 in *Drosophila*

**DOI:** 10.1093/nar/gks857

**Published:** 2012-09-18

**Authors:** Dmitriy Gurskiy, Anastasija Orlova, Nadezhda Vorobyeva, Elena Nabirochkina, Alexey Krasnov, Yulii Shidlovskii, Sofia Georgieva, Daria Kopytova

**Affiliations:** ^1^Department of Regulation of Gene Expression, Institute of Gene Biology, Russian Academy of Sciences, ^2^Centre for Medical Studies in Russia, University of Oslo, and ^3^Department of Transcription Factors, Institute of Molecular Biology, Russian Academy of Sciences, Vavilov Street 32, Moscow 119991, Russia

## Abstract

SAGA/TFTC is a histone acetyltransferase complex that has a second enzymatic activity because of the presence of a deubiquitination module (DUBm). *Drosophila* DUBm consists of Sgf11, ENY2 and Nonstop proteins. We show that Sgf11 has other DUBm-independent functions. It associates with Cbp80 component of the cap-binding complex and is thereby recruited onto growing messenger ribonucleic acid (mRNA); it also interacts with the AMEX mRNA export complex and is essential for *hsp70* mRNA export, as well as for general mRNA export from the nucleus. Thus, Sgf11 functions as a component of both SAGA DUBm and the mRNA biogenesis machinery.

## INTRODUCTION

Numerous studies demonstrate that a large amount of multiprotein coactivator complexes are organized into sophisticated systems to provide accurate and precise functioning of the RNA polymerase II (RNAP II) machinery. Moreover, different transcription stages are intimately connected to each other and to mRNP formation and nuclear export.

SAGA is a large multifunctional multisubunit coactivator complex that possesses histone acetyltransferase and histone deubiquitination enzymatic activities and is highly conserved through the evolution of eukaryotes (1). The histone acetyltransferase module of SAGA contains a Gcn5-catalytic subunit and operates by opening up the chromatin landscape to the binding of additional transcription factors, which results in PIC formation (2–4).

The deubiquitination module (DUBm) of yeast SAGA is composed of four subunits: Ubp8 (catalytic subunit), ySgf11, Sus1 and Sgf73. It has an ‘assembly lobe’ composed of the long N-terminal helix of ySgf11 and the ZnF-UBP domain of Ubp8 joined by Sus1, and a ‘catalytic lobe’ formed by the Sgf11 C-terminal zinc finger domain interacting with the Ubp8 catalytic domain near its active site. Sgf73 interacts with the two lobes of DUBm and binds it to SAGA (5,6).

Homologs of yeast DUBm components have been identified in higher eukaryotes (7–10): *Drosophila* Nonstop, Sgf11 and ENY2 are homologous to yeast Ubp8, Sgf11 and Sus1, respectively (11). They were shown to be components of SAGA (11), and the interaction between recombinant tagged Sgf11 and Nonstop was demonstrated experimentally (11,12). *Drosophila* Nonstop and Sgf11 have a role in H2B deubiquitination (11). A putative *Drosophila* ortholog of yeast Sgf73 was recently identified (13). However, the existence of an integrated DUBm in *Drosophila* has not yet been shown, and the functional organization of *Drosophila* DUBm is still a matter for discussion.

It is noteworthy that the Sus1 subunit of SAGA in yeast and humans is also a component of the messenger ribonucleic acid (mRNA) export complex named TREX-2. Sus1 is essential for general mRNA export and provides for ‘gating’ of active genes to the nuclear envelope (14,15). A similar role was demonstrated for *Drosophila* ENY2, which, together with Xmas-2, was found to be a component of the general mRNP export complex AMEX (a homolog of TREX-2). The *Drosophila* AMEX interacts with the nuclear pore complex (NPC) (16). Some SAGA complexes that are present at the nuclear periphery also interact with NPC. Both complexes are essential for the efficient expression of the *hsp70* gene on heat shock and for its anchoring to NPC (16).

In addition, Sus1/ENY2 is essential for transcription elongation both in yeast and *Drosophila* (15,17,18). ENY2 interacts with THO transcription elongation and mRNA export complex and is essential for mRNP biogenesis (18). Thus, Sus1/ENY2 has many ‘satellite’ partners in interactions, and all of them together coordinate transcription, mRNP biogenesis and export (16,17).

Although Sus1/ENY2 has been studied in detail, much less data are available on the function of the Sgf11 subunit of DUBm. In this study, we have shown that *Drosophila* Sgf11 is associated with Nonstop, ENY2 and Gcn5, suggesting that an integrated SAGA-associated DUBm exists in *Drosophila*. However, a moiety of Sgf11 is not associated with SAGA; it is recruited on the *hsp70* promoter in a RNA-dependent manner. Sgf11 (but not DUBm) binds to *hsp70* mRNA and is essential for its export, as well as for total mRNA export from the nucleus. Finally, we have shown that Sgf11 interacts with the cap-binding protein 80 (Cbp80) component of the cap-binding complex (CBC), and that Cbp80 knockdown interferes with Sgf11 recruitment on the *hsp70* promoter.

## MATERIALS AND METHODS

### Antibodies

Polyclonal antibodies against Sgf11 (full-length protein), Nonstop (1–160 and 166–496 aa fragments) and Cbp80 (552–799 aa fragment) were raised in our laboratory by immunizing rabbits with the corresponding His6-tagged protein fragments. Both anti-Nonstop antibodies recognized the same band in western blots, but experiments were nevertheless performed with the antibody against the N-terminal peptide. We also used polyclonal antibodies previously raised in our laboratory against ENY2 (19), Xmas-2 (16) and Thoc5 (18). Antibodies against Gcn5, Ada2b and Pol II were described elsewhere (16,20,21). All rabbit antibodies were affinity purified. An antibody against β-tubulin, obtained by M. Klymkowsky, was from the Developmental Studies Hybridoma Bank developed under the auspices of the National Institute of Child Health and Human Development and maintained at the Department of Biological Sciences, University of Iowa. The antibodies against NPC were from Abcam (ab24609). Cy3-conjugated goat anti-rabbit IgG (Amersham) and Alexa Fluor 488-conjugated goat anti-mouse IgG (Molecular Probes) were used as secondary antibodies.

### Purification of Sgf11-containing complexes and immunoprecipitation

Sgf11-containing complexes were purified from nuclear extracts of 0- to 12-h *Drosophila* embryos. The scheme of purification is described in results. The columns were equilibrated with HEMG buffer [25 mM HEPES-KOH at pH 7.6, 12.5 mM MgCl_2_, 0.1 mM ethylenediaminetetraacetic acid (EDTA), 10% glycerol and 1 mM DTT] containing 150 mM NaCl (HEMG-150). Immunoaffinity purification was performed on a column with immobilized anti-Sgf11 antibodies, with the unbound protein washed out with a HEMG-500 buffer containing 0.1% NP-40. Details of the purification procedure and MALDI-TOF MS experiments were described previously (22,23). The Superose 6 column was calibrated with an HMW Calibration Kit (GE Healthcare). The void volume of the column was 7.0 ml, and the volume of each fraction was 0.5 ml. The preparation of embryonic nuclear extracts and immunoprecipitation were performed as described previously (19). Proteins from *Drosophila* Schneider 2 (S2) cells were separated into the nuclear and cytoplasmic fractions using a lysis buffer (20 mM HEPES at pH 7.8, 2.5 mM MgCl_2_, 100 mM NaCl and 1 mM DTT) containing the complete protease inhibitor cocktail (Roche) and phosphatase inhibitor cocktails 1 and 2 (Sigma) (24,25). Before immunoprecipitation, the extract was treated with DNase I (USB, 0.6 U/ml) and RNase (ribonuclease) (Stratagene, 10 U/ml).

**Figure 1.**
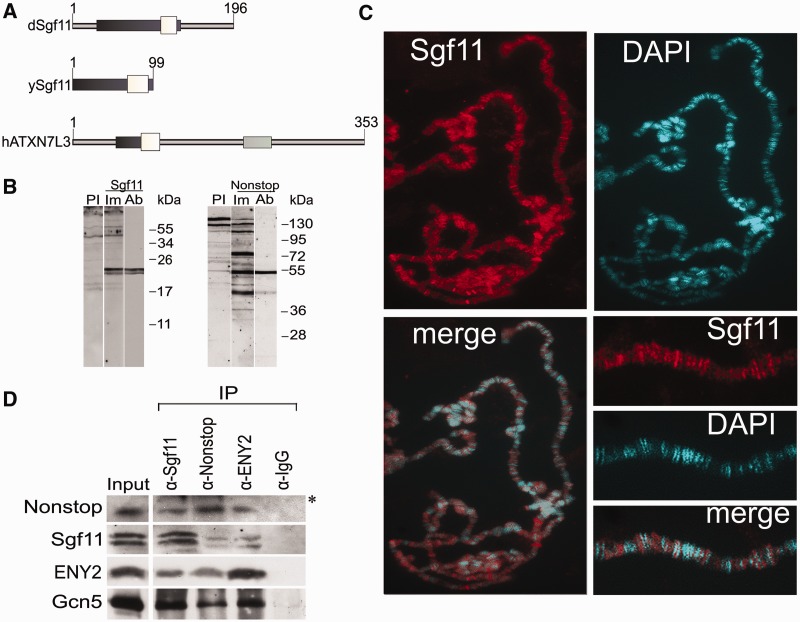
Nonstop, ENY2 and the moiety of Sgf11 form the *Drosophila* DUBm that interacts with SAGA. (**A**) Sgf11 structure and homology in different species (*Drosophila*, yeast and human). White boxes show homologous Sgf11-related domains; dark gray boxes, Sgf11 zinc finger-containing evolutionarily conserved regions and light gray boxes, the SCA7 domain, which is present only in the human protein. (**B**) Affinity-purified polyclonal antibodies raised in rabbits specifically recognize Sgf11 and Nonstop in nuclear extract from *Drosophila* embryos in western blot analysis. Lanes were stained with preimmune sera (PI), immune sera (Im) and affinity-purified polyclonal (Ab) antibodies are shown. (**C**) Sgf11 binds to numerous sites on polytene chromosomes from *Drosophila* salivary glands. Chromosomes stained with anti-Sgf11 antibodies and DAPI and merged images and scaled-up fragments of chromosomes are shown. (**D**) Sgf11 interacts with DUBm subunits and the Gcn5 component of SAGA in nuclear extract from *Drosophila* embryos in co-immunoprecipitation experiments (IP). Antibodies against Sgf11, Nonstop, ENY2 and Gcn5 or preimmune serum (PI) were used. Bands indicated with an asterisk correspond to antibodies.

### ChIP and qPCR analysis

ChIP assay was performed according to the published protocol (23). Deoxyribonucleic acid (DNA) was cross linked (1% FA, 15 min) and sheared into fragments of about 300 bp in size. Approximately 3 × 10^6^ cells and 10 µg of an antibody were taken for one experiment. Thereafter, ChIP DNA was recovered; DNA was analyzed by qPCR using the MiniOpticon system (Bio-Rad) (primers are available on request). The amount of proteins was measured in the promoter region within the *hsp70* gene, namely, at position −43 relative to the transcription start point. In each case, measurements were made in at least five replications, and the mean value was calculated. The level of corresponding protein determined at three points in intergenic spacer sequences was taken as a baseline. In a special experimental series, RNase A (Fermentas, 30 µg per experiment) was added 30 min before incubation with antibodies.

### RNA immunoprecipitation (RIP)

Co-immunoprecipitation of *hsp70*, *Ras* and *tubulin* mRNAs was performed as described previously (24,25) from the lysate of S2 cells kept at normal temperature or exposed to heat shock at 37°C for 20 min. No cross-linking reagent was used. For immunoprecipitation, the lysate was incubated for 1 h at 4°C with the antibody–protein A Sepharose beads in NT2 buffer (50 mM Tris–HCl at pH 7.4, 150 mM NaCl, 1 mM MgCl_2_ and 0.05% NP-40) containing 4 U/ml RiboLock (Fermentas), 1% bovine serum albumin and 1 mg/ml salmon sperm DNA. The beads were then washed and treated with the TRI Reagent (Ambion) to extract RNA. Reverse transcription was performed with an oligo-dT primer. The level of *hsp70* mRNA was measured by qPCR using primers 5′-TTGGGCGGCGAGGACTTTG-3′ and 5′-GCTGTTCTGAGGCGTCGTAGG-3′. Each experiment was performed in at least three replications, and the mean value was calculated.

### FISH

Staining for total polyA RNA was carried out as described previously (26) using a 50-bp Cy3-labeled oligo-dT probe. For RNA FISH of individual genes, S2 cells were fixed on glass slides (27), and specific probes were synthesized with the following primers: *hsp70* (a 2405-bp fragment), primers 5′-CGACATACTGCTCTCGTTGGTTC-3′ and 5′-AGCTAAAATCAATTTGTTGCT AACTT-3′; *Ras2* (a 549-bp fragment), primers 5′-ATGCAAACGTACAAACTGGTGG-3′ and 5′-GCCCTTCTTCTTGTAATCCTGC-3′ and *tubulin* (a 1059-bp fragment), primers 5′-AT GTTAATCGGAGCTCGGCC-3′ and 5′-CCATTCGACGAAGTAGGAGCTG-3′. The probes were labeled with biotinylated-16-dUTP using Biotin-Nick Translation Mix (Roche). After purification, a labeled probe was resuspended in a hybridization buffer (50% formamide, 2× SSC, 10% dextran sulfate, 1% Tween-20, 1 mg/ml yeast transfer RNA and 0.5 mg/ml sonicated salmon sperm DNA) to a final concentration of 2–5 ng/ml. A 10 -µl aliquot of the probe was applied onto the preparation, denatured at 75°C for 5 min and hybridized overnight at 37°C. The preparations were washed in 50% formamide, 2× SSC at 45°C and 0.1× SSC at 55°C, and the probes were visualized using three layers of Alexa 488-conjugated avidin (Invitrogen) and biotin-conjugated antiavidin D antibody (Vector Laboratories).

### *Drosophila* cell culture, RNAi knockdown and transfection experiments

*Drosophila* S2 cells were cultured at 25°C in Schneider’s insect medium (Sigma) containing 10% fetal bovine serum (HyClone). RNAi experiments followed the published protocol (28). We used 15–20 mg of dsRNA per 1–3 × 10^6^ cells; dsRNA was synthesized with an Ambion MEGAscript T7 kit. The expression of the target genes was measured by qPCR and western blot analysis. dsRNA corresponding to a fragment of green fluorescent protein (GFP) was used as a negative control. The following primers were used for the synthesis: Sgf11 full, 5′-CGACTCACTATAGGGAGACGACGACAACAGGAGCTCAG-3′ and 5′-CGACTCACTATAGGGAGAGAGCCGTTGTTCCTTGAGTTC-3′; Sgf11 1_1, 5′-CGACTCACTATAGGGAGATCAACGGCCAAAAAGCCAAT-3′ and 5′-CGACTCACTATAGGGAGAGAGCCGTTGTTCCTTGAGTTC-3′; Sgf11 1_2, 5′-CGACTCACTATAGGGAGACGACGACAACAGGAGCTCAG-3′ and 5′-CGACTCACTATAGGGAGAATGCCGAATATGTCCAGGTT-3′; Nonstop, 5′-CGACTCACTATAGGGAGA AGCACGAAAGCAAGAGCAAC-3′ and 5′-CGACTCACTATAGGGAGAGGATTTTCTTA CTCGTATTCCAGC-3′; Cbp80, 5′-CGACTCACTATAGGGAGACTCACGACACGG AGGA TGAG-3′ and 5′-CGACTCACTATAGGGAGAGGGCAGACTCCTTCTTCTCGT-3′; ENY2, 5′-GAATTAATACGACTCACTATAGGGAGCACTTCCGGCGCAGTTGATC-3′ and 5′-GAATTAATACGACTCACTATAGGGAGGATTCGTCCTCTGGCTCA-3′ and Xmas-2, 5′-GAATTAATACGACTCACTATAGGGAGAATGACCTGCACCGTAAG-3′ and 5′-GAATTAATACGACTCACTATAGGGAGACCGGTTGTAGTTCATAG-3′. Transient transfection of S2 cells was performed using Effectin (Qiagen).

### Constructs for Cbp80, Cbp20 and Sgf11 expression

The HA tag followed by a *Not*I site was cloned in pAc5.1/V5-His B (Invitrogen). The coding sequences of Cbp80 and Cbp20 with an upstream *Not*I site were inserted into the construct in frame with the HA tag. The coding sequence of Sgf11 was cloned into the construct in frame with the FLAG tag, which was cloned in pAc5.1/V5-His B (Invitrogen).

### Immunostaining

Immunostaining of S2 cells and polytene chromosomes was performed as described previously (22,29) using rabbit anti-Sgf11, rabbit anti-Nonstop and corresponding secondary antibodies (Molecular Probes). The results were analyzed under a TCS SP2 confocal microscope or a DMR/HC5 fluorescence microscope (Leica) with an HCX PZ Fluotar ×100/1.3 objective lens and recorded using a Leica DC350 F digital camera.

## Results

### *Drosophila* Sgf11 is a component of SAGA DUBm; a moiety of Sgf11 is not associated with SAGA

The Sgf11 homologs from yeast, *Drosophila* and humans share an Sgf11-related domain and a zinc finger-containing evolutionarily conserved region (Figure 1A). *Drosophila* Sgf11, compared with the yeast protein, has N- and C-terminal extensions, and human ATXN7L3 contains an SCA7 domain, which is absent in other proteins. To characterize *Drosophila* Sgf11, antibodies against this protein were raised in two rabbits. Rabbit antibodies were also obtained against the Nonstop subunit of DUBm. Antibodies against the ENY2 component were described previously (19). Affinity-purified antibodies against Sgf11 and Nonstop efficiently recognized the respective proteins in the nuclear extract from *Drosophila* embryos, with the molecular weights of respective bands corresponding to the expected values (Figure 1B). Both antibodies against Sgf11 detected the same two closely migrating bands in protein extracts from *Drosophila* embryos (Supplementary Figure S1A) or S2 cells. Both Sgf11 bands equally reacted with anti-Sgf11 antibodies and were equally depleted after Sgf11 RNAi knockdown (Figures 1D, 3C and 5B; Supplementary Figure S2D). The quantitative ratio of these bands varied between different extracts, suggesting that Sgf11 may have a modification that is unstable during extract preparation.

**Figure 2.**
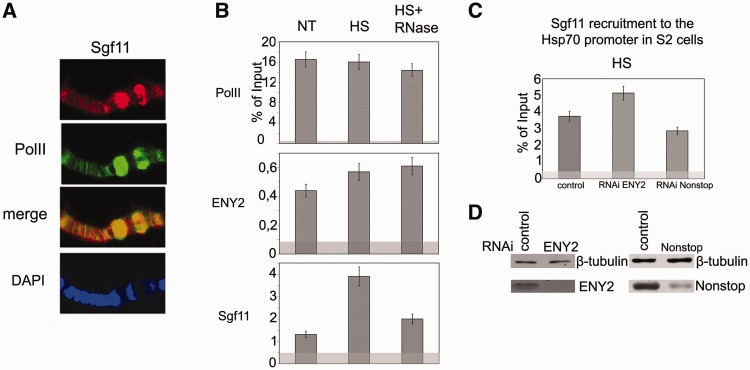
Sgf11 is present on the *hsp70* promoter region. (**A**) Sgf11 is present in *hsp70* puffs (87 A, 87B) on polytene chromosomes of *Drosophila* larvae under heat shock. Chromosomes were co-stained with antibodies against Sgf11, Pol II and DAPI. (**B**) Sgf11 occupancy of the *hsp70* promoter increased after heat shock (HS) and significantly decreased after RNase treatment. The presence of Sgf11, Pol II and ENY2 was analyzed by ChIP before (NT) and after heat shock (HS). The results of ChIP are shown as a percentage of input. Here, and in Figures 2C and 6A–G, light gray shading indicates the background (baseline) level determined as the average of measurements in three noncoding sequences (see ‘Materials and Methods’ section). (**C**) Effects of ENY2 and Nonstop RNAi knockdown on Sgf11 recruitment on the *hsp70* promoter under heat shock conditions (HS) according to the results of ChIP (shown as a percentage of input). Cells treated with GFP dsRNA were used as control. (**D**) The efficiency of ENY2 and Nonstop RNAi knockdown in experiments shown on Figure 3 C as verified by western blot analysis. Cells were treated with GFP dsRNA (control) or dsRNA corresponding to Sgf11 and Nonstop. Tubulin was used as loading control.

The immunostaining of *Drosophila* larval salivary gland polytene chromosomes showed that Sgf11 and Nonstop bound to numerous interband sites, which was indicative of their association with actively transcribed chromatin (Figure 1C, Supplementary Figure S1B).

Recent studies have revealed an interaction between recombinant tagged Sgf11 and Nonstop in *Drosophila* S2 cells (11,12). Accordingly, antibodies against Sgf11 proved to co-precipitate Nonstop from the nuclear extract of *Drosophila* embryos. Moreover, antibodies against either Sgf11 or Nonstop co-immunoprecipitated ENY2 and the Gcn5 subunit of SAGA (Figure 1D), confirming the existence of SAGA-associated DUBm in *Drosophila*. As expected, ENY2 as a component of other complexes associated with mRNP biogenesis and export was not completely depleted from the extract by antibodies against Sgf11 and Nonstop. It is noteworthy that a certain amount of Sgf11 also remained in the extract after treatment with antibodies against Nonstop or ENY2, suggesting that this protein may occur not only within the DUBm.

### Sgf11 is recruited onto the *hsp70* promoter in an RNA-dependent manner after transcription activation

The function of Sgf11 in gene expression was analyzed using the *Drosophila hsp70* gene, with regard to our previous data that the SAGA complex participates in its transcription during heat shock (20). The antibodies against Sgf11 strongly stained *hsp70* puffs on *Drosophila* larval polytene chromosomes after heat shock, indicating that Sgf11 also participates in transcription of this gene (Figure 2A).

**Figure 3.**
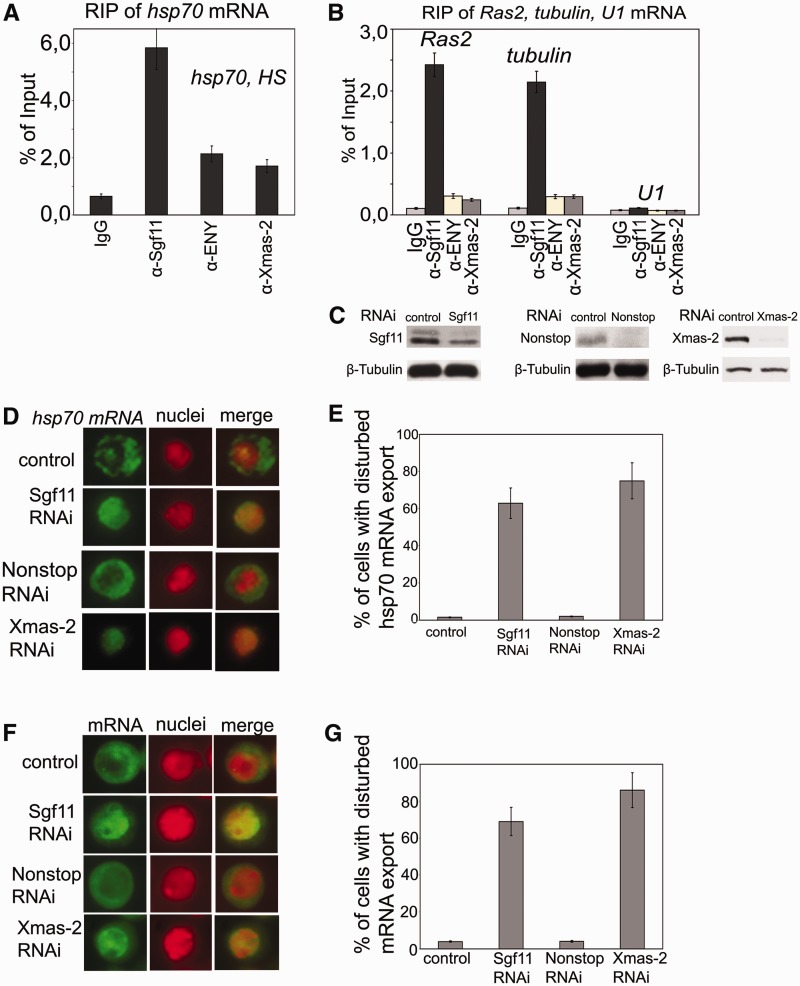
Sgf11 is associated with mRNAs of several genes, and its RNAi knockdown interferes with general mRNA export. (**A**) RIP experiments with *hsp70* mRNA after heat shock were performed using antibodies against Sgf11 or components of the mRNA-interacting AMEX complex (ENY2, Xmas-2); nonimmune IgG was used as control. The results are shown as a percentage of input. (**B**) Sgf11 binds to mRNAs of *Ras* and *tubulin* genes under normal conditions. The *U1* snRNA was used as a control. Antibodies used in RIP experiments were the same as in Figure 3 A. The results are shown as a percentage of input. (**C**) The level of Sgf11, Nonstop and Xmas-2 knockdown in experiments shown in Figures 3D–3G as estimated by western blot analysis in cells treated with GFP dsRNA (control) or dsRNA corresponding to Sgf11 and Nonstop. Tubulin was used as a loading control. (**D**) RNAi knockdown of Sgf11, but not Nonstop, interferes with *hsp70* mRNA export after heat shock. Cells were treated with GFP dsRNA (control) or dsRNA corresponding to Sgf11 and Nonstop. Xmas-2 RNAi knockdown was performed as a positive control. Representative examples of the distribution of *hsp70* mRNA (green staining) and cell nuclei (red staining) and corresponding merged images are shown for control cells and cells after Sgf11, Nonstop or Xmas-2 knockdown (magnification, ×1000). The *hsp70* transcript was detected by RNA FISH using an Alexa 488-labeled probe; the nuclei were stained with DAPI. The images were recolored in Photoshop for better visualization. (**E**) Quantitative presentation of the results of experiments shown on Figure 3D. Bars show the percentage of cells with disturbed *hsp70* mRNA nuclear export (about 200 cells per RNAi experiment were examined). (**F**) RNAi knockdown of Sgf11, but not Nonstop, interferes with general mRNA export. Cells were treated with GFP dsRNA (control) or dsRNA corresponding to Sgf11 and Nonstop. Xmas-2 RNAi knockdown was performed as a positive control. Representative examples of the distribution of mRNA (green staining) and cell nuclei (red staining) and corresponding merged images are shown for control cells and cells after Sgf11 or Nonstop knockdown (magnification, ×1000). RNA FISH was carried out using a Cy3-labeled oligo(dT) probe to identify poly(A) RNA. The nuclei were stained blue with DAPI. The images were recolored in Photoshop for better visualization. (**G**) Quantitative presentation of the results of experiments shown in Figure 3 F. Bars show the percentage of cells with disturbed hsp70 mRNA nuclear export (about 200 cells per RNAi experiment were examined).

**Figure 4.**
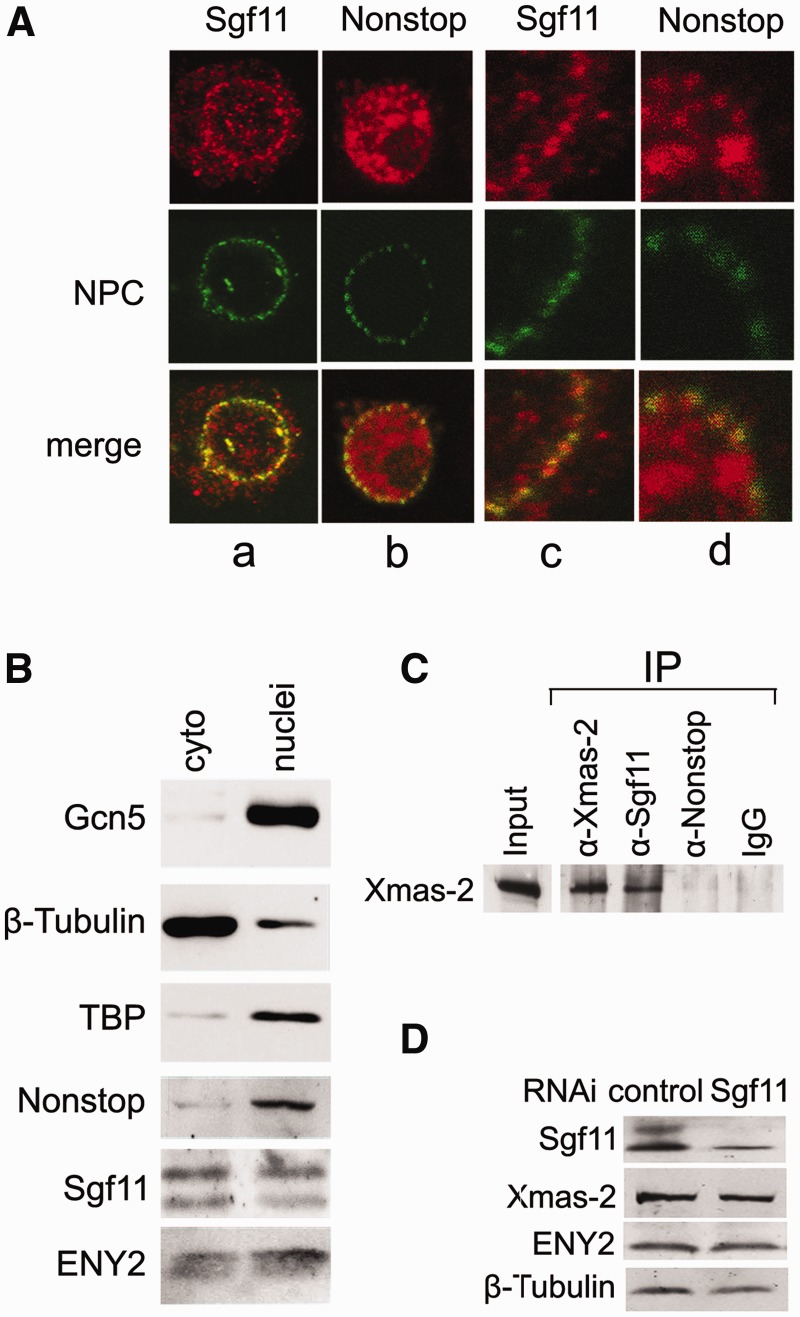
Sgf11 is distributed between the nucleus and cytoplasm, co-localizes with NPC and interacts with AMEX. (**A**) Immunostaining of *Drosophila* S2 cells with antibodies against Sgf11 or Nonstop (red) and NPC (green) and merged images (magnification, ×1000). Panels c and d show cell fragments at higher magnification (×10000). (**B**) Sgf11 and Nonstop distribution in S2 cell nuclei and cytoplasmic extracts analyzed by western blotting. Equal amounts of extracts were loaded, and the blot was stained with antibodies against indicated proteins. Antibodies against TBP and β-tubulin were used to confirm that the cytoplasmic fraction was not contaminated with nuclear proteins, and vice versa. (**C**) Sgf11, but not Nonstop, interacts with Xmas-2, a component of the mRNA export complex AMEX, in co-immunoprecipitation from nuclear extract of *Drosophila* embryos. Antibodies against Xmas-2, Sgf11, Nonstop and IgG (control) were used. Western blot was stained with antibodies against Xmas-2. (**D**) Sgf11 RNAi knockdown does not affect the level of AMEX components in S2 cells. The presence of the indicated factors was analyzed in cells treated with control nonspecific dsRNA (GFP) or corresponding dsRNA. Tubulin was used as loading control. The levels of corresponding proteins in sham-treated cells (left lanes) or in cells after RNAi treatment (right lanes) are shown.

Next, ChIP assay was used to study the occupancy of the *hsp70* promoter by Sgf11 and other DUBm subunits before and after gene activation. In accordance with previous data (30–33), approximately similar amounts of stalled Pol II were detected on the *hsp70* promoter before and after heat shock (Figure 2B). The level of ENY2 and Nonstop components of DUBm moderately increased after heat shock (Figure 2B, Supplementary Figure S1C).

As expected, Sgf11 as a DUBm component was also detected on the *hsp70* promoter before and after gene activation. However, unlike in cases of ENY2 and Nonstop, the amount of Sgf11 on the promoter strongly increased after heat shock (Figure 2B). This result suggests that an additional amount of ‘free’ Sgf11 (not incorporated in DUBm) is recruited to the gene on transcription activation.

To verify whether Sgf11 association with *hsp70* depends on nascent mRNA, we treated chromatin with RNase as described previously (34) to find out whether this treatment would have an effect on the results of ChIP. The Pol II level remained unchanged on RNase treatment, and the ENY2 peak on the promoter was also insensitive to RNase, in agreement with our previous results (18). The same was observed for Nonstop (Supplementary Figure S1C). Unexpectedly, RNase treatment proved to significantly reduce the amount of Sgf11, approximately to the level found on the promoter before heat shock (Figure 2B). This fact suggested that RNA is necessary for Sgf11 recruitment to the *hsp70* promoter.

Next, we tested whether Sgf11 recruitment onto the promoter during *hsp70* activation depends on other DUBm components (Figure 2C). The differences in the Sgf11 level after RNAi knockdown of ENY2 or Nonstop were not significant. The RNAi knockdown of ENY2 slightly increased the Sgf11 level. This may be explained by a better accessibility of Sgf11 to antibodies, as ENY2 within DUBm may partially screen it. The RNAi knockdown of Nonstop resulted in only a slight decrease in the Sgf11 level. Hence, it appears that the Sgf11 moiety not incorporated in DUBm is recruited to the gene after transcription activation because of the interaction of this protein with nascent mRNA.

### Sgf11 binds to *hsp70* mRNA and plays an essential role in *hsp70* mRNA export after heat shock and in total mRNA export

The results of ChIP assay indicating that Sgf11 interacts with nascent *hsp70* mRNA were verified by analyzing this interaction in RNA immunoprecipitation (RIP) experiments with the lysate of heat-shocked S2 cells. Antibodies against Sgf11 precipitated a significant amount of *hsp70* mRNA (Figure 3A). Antibodies against Xmas-2 and ENY2 components of the general mRNA export complex AMEX also co-precipitated *hsp70* mRNA. In addition, all these antibodies co-precipitated mRNAs of two other genes (*Ras2* and *tubulin*) from S2 cells lysate (Figure 3B). However, no interaction of Sgf11, Xmas-2 or ENY2 with *U1* snRNA was observed (Figure 3B), confirming the specificity of the experiments.

Next, we tested whether Sgf11 plays a role in mRNA export. To this end, we used RNA FISH to evaluate the effect of Sgf11 RNAi knockdown on the distribution of *hsp70* mRNA between the nucleus and cytoplasm under heat shock conditions. The results showed that the nuclear export of the *hsp70* transcript was significantly disturbed by Sgf11 knockdown (Figure 3D), with the proportion of cells with defects in mRNA export reaching 70% (Figure 3E). In comparison, RNAi knockdown of the Xmas-2 component of the AMEX general mRNA export complex caused defects in 90% of cells (Figure 3D and E). The observed effect was not due to disturbances in *hsp70* transcription, as the *hsp70* mRNA level remained almost unchanged after Sgf11 knockdown (Supplementary Figure S2A). This effect was also independent of DUBm because RNAi knockdown of Nonstop had no influence on mRNA export (Figure 3D and E). We also observed that Sgf11 RNAi disturbed the nuclear export of *Ras2* and *tubulin* mRNA at normal temperature (data not shown).

Thus, mRNA export defects were observed on three different randomly chosen mRNAs (one inducible gene and two housekeeping genes). In addition, it was demonstrated previously that deletion of the Sgf11 coding gene enhanced mRNA export defects in mutants with Sus1/ENY2 deletion (35). Thus, we suggested that Sgf11 may have a general role in mRNA export and investigated the effect of Sgf11 knockdown on the distribution of total mRNA between nuclei and cytoplasm using an oligo-dT probe. The results of these experiments demonstrated that Sgf11 was essential for total mRNA export, as a major proportion (about 65%) of Sgf11 RNAi knockdown cells had defects in this process (Figure 3F and G). Moreover, the same effect was observed when RNAi knockdown was performed with different Sgf11 fragments (Supplementary Figure S2C). Xmas-2 RNAi caused export defects in about 75% of knockdown cells. Unlike in experiments with Sgf11, Nonstop RNAi had no influence on mRNA distribution between the nucleus and cytoplasm (Figure 3F and G), suggesting that DUBm is not involved in the observed effect.

### Sgf11 is distributed between the nucleus and cytoplasm, co-localizes with NPC and interacts with the AMEX mRNA export complex

For comparision the intracellular distribution of Sgf11 and DUBm, S2 cells were immunostained with affinity-purified antibodies against Sgf11 or Nonstop and co-stained with antibodies against NPC (Figure 4A). Both Sgf11 and Nonstop were distributed in the nucleus in a dot-like pattern and co-localized with NPC at the nuclear periphery (Figure 4A), as it was shown previously for the other subunits of the SAGA complex (16).

**Figure 5.**
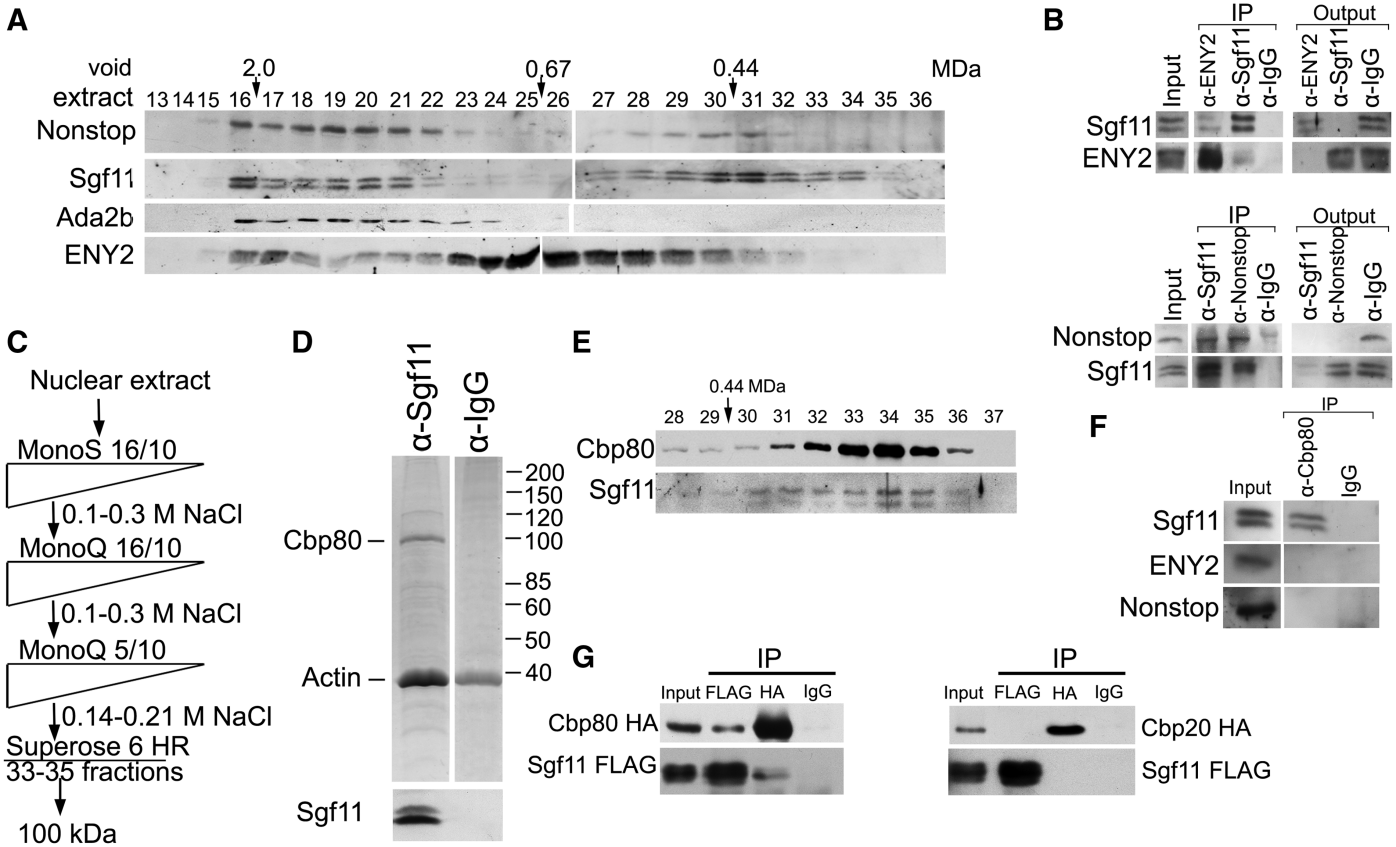
Sgf11 is present in several fractions of nuclear extract from *Drosophila* embryos and is associated with Cbp80 in a separate complex. (**A**) Sgf11 and Nonstop are present in low molecular weight fractions that do not contain SAGA complex. *Drosophila* embryonic extract treated with DNase I and RNase was fractionated on a Superose 6 gel filtration column. The fractions were analyzed for the presence of Sgf11, Nonstop, ENY2 and ADA2b (as a marker of SAGA distribution) by western blot analysis. Fraction numbers and the void volume are indicated. The distribution of ENY2, which has several characteristic peaks, testifies to the quality of fractionation. (**B**) Sgf11, Nonstop and ENY2 interact in low molecular weight fractions. Co-immunoprecipitation from the nuclear extract was performed with antibodies against Sgf11, Nonstop and ENY2 components of DUBm or with a preimmune serum (PI) in low molecular weight fractions 28–31. Equal amounts of the extract (Inp) and precipitated proteins (IP) were analyzed. (**C**) Scheme for purification of Sgf11-containing complexes. At each step, proteins were eluted with a NaCl gradient, and the fractions were analyzed for the presence of Sgf11 by western blotting. The peak Sgf11-containing fractions (the corresponding NaCl concentrations are indicated) were collected and loaded onto the next column. After Superose 6 fractionation, the material was loaded onto an immunosorbent with antibodies against Sgf11, washed and eluted with acid glycine. (**D**) Preparation of the Sgf11-containing complex purified from fractions 33–35 (∼100 kDa). Proteins eluted from the immunosorbent were resolved by 9% SDS-PAGE, stained with Coomassie and analyzed by mass spectrometry. The control immunoprecipitation of the same material with IgG is shown on the right. The bottom panels show western blot analysis of the Sgf11-containing preparation and the control immunoprecipitation for the presence of Sgf11. (**E**) The migration profiles of Cbp80 and Sgf11 on the Superose 6 column at the final purification step coincide with each other. (**F**) Co-immunoprecipitation experiments with nuclear extract of *Drosophila* embryos reveal no interactions between Cbp80 and ENY2 or Nonstop DUBm components. (**G**) Recombinant Sgf11 interact with Cbp80 but not with Cbp20. FLAG-tagged Sgf11 was co-expressed with HA-tagged Cbp80 or HA-tagged Cbp20 in transiently transfected S2 cells. Immunoprecipitation was performed with anti-FLAG or anti-HA antibodies or with IgG. Western blot was stained with anti-FLAG of anti-HA antibodies. About 10% of the input and 50 % of the precipitate were loaded onto the gel.

Moreover, Nonstop was detected only in the nuclei, whereas dot-like positive staining for Sgf11 was also observed in the cytoplasm, confirming that a certain amount of this protein is not associated with DUBm or SAGA. Such a distribution resembled that of ENY2 and Xmas-2 components of the AMEX mRNA export complex, which were previously also shown to have a dot-like staining pattern in the cytoplasm (16,18).

To verify these results, S2 cells were fractionated into the nuclei and cytoplasm, and the proteins contained in these fractions were analyzed by western blotting (Figure 4B). The Nonstop component of the DUBm, similar to Gcn5, was detected only in the nuclear fraction, whereas Sgf11 and ENY2 were almost evenly distributed between the nuclei and cytoplasm (Figure 4B), confirming the results of the immunostaining.

As Sgf11 interacted with mRNA in RIP experiments, had an effect on total mRNA export and co-localized with NPC, we used co-immunoprecipitation to test whether Sgf11 interacted with the AMEX general mRNA export complex. The results showed that anti-Sgf11 (but not anti-Nonstop) antibodies co-precipitated a certain amount of the Xmas-2 component of AMEX (Figure 4C). This evidence for the interaction of Sgf11 with AMEX is in line with a role for Sgf11 in general mRNA export. We also verified that the Xmas-2 and ENY2 levels in the cells remained unchanged on Sgf11 RNAi knockdown and, therefore, could not be responsible for the observed defects in mRNA export (Figure 4D). It is more probable that the depletion of Sgf11 interferes with its interaction with AMEX, thereby disturbing the effective export of mRNP particles.

### Sgf11 is present in several complexes in *Drosophila* embryo nuclear extract and is associated with the Cbp80 independently of DUBm

Our data that Sgf11 participates in *hsp70* transcription and is also involved in total mRNA export suggested that it may be a component of not only SAGA but also of some other protein complexes. To identify such Sgf11-containing complexes, the nuclear extract from *Drosophila* embryos was fractionated on a Superose 6 gel filtration column, and the fractions were tested for the presence of DUBm by western blotting with antibodies against Sgf11 and Nonstop (Figure 5A). To mark the SAGA complex, antibodies against the SAGA-specific component Ada2b were used.

The distribution of ENY2 was characterized by several peaks, in agreement with our previous data (16,18). Both Nonstop and Sgf11 were detected in high molecular weight Superose 6 fractions, where their profile coincided with Ada2b, suggesting that DUBm in these fractions is associated with the SAGA complex. In addition, the second broad peak of Nonstop was observed in low molecular weight fractions 28–31 (Figure 5A), where Sgf11 was also detected. We supposed that low molecular weight fractions could contain a separate DUBm and performed a co-immunoprecipitation experiment to test this assumption (Figure 5B). The results showed that antibodies against Nonstop or ENY2 co-precipitated Sgf11, and vice versa, indicating that DUBm as a separate molecule was indeed present in these fractions (Figure 5B).

However, antibodies against Nonstop and ENY2 failed to completely deplete Sgf11 from the extract (Figure 5B), suggesting that low molecular weight fractions contained Sgf11 that was not incorporated in DUBm. This result was in agreement with different migration patterns of Nonstop and Sgf11 in low molecular weight fractions. Sgf11 was found in Superose 6 fractions 33–35, where Nonstop was either absent or its amount was below the detection limit. ENY2 was detected in insignificant amounts and only after prolonged exposure.

To study Sgf11-associated proteins from fractions 33–35 and the Sgf11-containing complex, we performed their stepwise chromatographic purification from the embryonic nuclear extract by the scheme shown in Figure 5C. At the final step, partially purified Sgf11-containing fractions from MonoQ were loaded onto a Superose 6 gel filtration column, fractions corresponding to the LMW peak (fractions 33–35, molecular weight of about 100 kD) were collected, and the proteins associated with Sgf11 were co-precipitated with affinity-purified antibodies against Sgf11, resolved in sodium dodecyl sulphate-polyacrylamide gel electrophoresis (SDS-PAGE) and identified by MALDI-TOF MS.

Unexpectedly, we found Cbp80, a component of CBC, to be associated with Sgf11 (Figure 5D). The antibodies also co-precipitated actin, but this interaction appeared unspecific because the same band was observed in the control reaction with nonimmune IgG (Figure 5D). To confirm these results, antibodies against Cbp80 were raised in rabbits and affinity purified (Supplementary Figure S3A). Using these antibodies, we found that Cbp80 and Sgf11 had similar migration profiles in low molecular weight fractions from Superose 6 at the final purification step (Figure 5E). Experiments on co-immunoprecipitation from the embryonic extract confirmed the Cbp80–Sgf11 interaction, suggesting that these proteins may form an individual complex. However, no interaction was observed between Cbp80 and ENY2 or Nonstop (Figure 5F). Thus, it appears that DUBm does not interact with Cbp80.

The interaction between Sgf11 and Cbp80 could be mediated by other proteins, such as the Cbp20 component of CBC, which could be lost during the complex purification procedure because of its small molecular weight. To test for the interaction of Sgf11 with components of CBC, FLAG-tagged Sgf11 was co-expressed with HA-tagged Cbp80 or HA-tagged Cbp20 in S2 cells (Figure 5G). In immunoprecipitation experiments, antibodies against FLAG co-precipitated HA-tagged Cbp80, and vice versa, but no interaction of HA-tagged Cbp20 and FLAG-tagged Sgf11 was observed. These results provide evidence for the direct interaction of Sgf11 with Cbp80.

### Cbp80 knockdown interferes with Sgf11 recruitment onto the *hsp70* promoter

**Figure 6.**
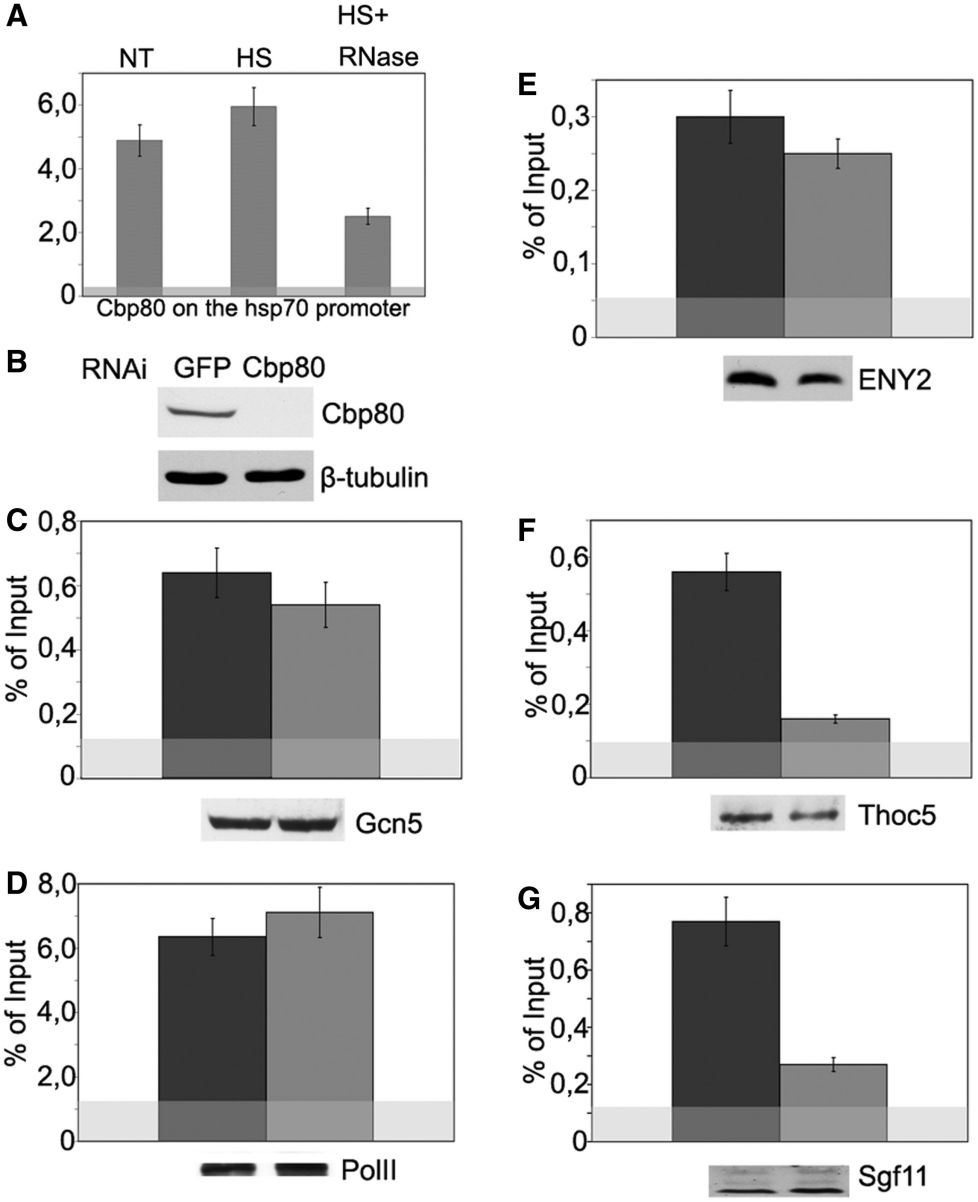
Sgf11 recruitment onto the *hsp70* promoter region upon transcription activation depends on Cbp80. (**A**) Cbp80 occupancy of the *hsp70* promoter as studied by ChIP. Chromatin was prepared before heat shock (NT), after heat shock (HS) or after heat shock with RNase treatment (HS+RNase). The results of ChIP are shown as a percentage of input. (**B**) Specificity of Cbp80 knockdown as tested by western blot analysis. The presence of Cbp80 was analyzed in cells treated with GFP dsRNA (control) or dsRNA. Tubulin was used as loading control. (**C–G**) The effect of Cbp80 RNAi knockdown on the recruitment of indicated proteins onto the *hsp70* promoter after heat shock as studied by ChIP. Western blotting was used to confirm that Cbp80 knockdown did not affect the total level of test proteins in the cells (the results are shown below each figure).

To evaluate the functional significance of the Sgf11–Cbp80 interaction, we first checked whether Cbp80 could be detected on the *hsp70* promoter. The promoter-associated peak of Cbp80, which increased after heat shock, was detected in ChIP experiments (Figure 6A). The association of Cbp80 with the promoter was RNA dependent and significantly decreased upon RNase treatment, confirming that Cbp80 is recruited in an RNA-dependent manner. The significant amount of Cbp80 detected in the absence of heat shock may be explained by its recruitment by stalled Pol II, as the interaction between Cbp80 and Pol II was demonstrated previously (36,37).

The fact that an additional pool of Sgf11 is recruited onto *hsp70* in an RNA-dependent manner under heat shock conditions suggests that promoter occupancy by Sgf11 may be dependent on Cbp80. To test this possibility, Cbp80 RNAi knockdown was performed in S2 cells, and the presence of Pol II, Sgf11 and several SAGA components on the *hsp70* promoter was analyzed by ChIP. The level of the THOC5 component of the THO complex was also measured as THO was previously shown to interact with nascent mRNA through binding to Cbp80 (38). Western blot analysis demonstrated that the overall level of the aforementioned factors in the cell was not affected by Cbp80 depletion (Figure 6C–G, lower panels). The Cbp80 RNAi knockdown had no significant effect either on the level of *hsp70* transcription (Supplementary Figure S3B) or on the level of the promoter-bound Gcn5 and Pol II (Figure 6C and D).

In accordance with the role of Cbp80 in the recruitment of the THO complex, the level of THOC5 decreased by more than half on Cbp80 RNAi knockdown (Figure 6F). The same was true of the Sgf11 level (Figure 6G), suggesting that Cbp80 is also essential for the recruitment of this protein. Importantly, the level of ENY2 was not affected by Cbp80 RNAi (Figure 6E). According to previous data on yDUBm structure, Sus1 is associated with yDUBm through ySgf11 (1,12). Therefore, the presence of ENY2 indicates that DUBm-associated Sgf11 remains intact after CBP depletion. In the aggregate, these results indicate that Cbp80 is important for the promoter recruitment of Sgf11 that is not associated with DUBm.

## DISCUSSION

The results presented above show that *Drosophila* Sgf11, a component of the DUBm of SAGA, has a function in general mRNA export. This function is supposed to be realized through the interaction of Sgf11 with the Cbp80 subunit of CBC and with the mRNA export complex AMEX. Our data also demonstrate that the Nonstop subunit of DUBm is not essential for mRNA export. These results correlate with data obtained on yeast, where no function in mRNA export for Upb8, a homolog of Nonstop, was found (35). Interestingly, in line with the results obtained on *Drosophila*, yeast Sgf11 has a role in mRNA export as the deletion of the Sgf11 coding gene has been shown to enhance the export deficiency phenotype in mutants with Sus1/ENY2 deletion (35).

In the first stage of this study, we demonstrated that endogenous *Drosophila* ENY2, Sgf11 and Nonstop form an integrated DUBm associated with SAGA. In agreement with the previous finding on yeast DUBm (35), *Drosophila* DUBm may partially dissociate from SAGA under certain conditions.

Next the function of *Drosophila* Sgf11 was studied on the *hsp70* gene model. Sgf11 was detected on the *hsp70* promoter, and its association with the promoter proved to be RNA dependent (at least partially), unlike that of ENY2 or Nonstop. In line with this fact, we found that Sgf11 was associated with *hsp70* mRNA and with mRNAs of two other genes (*Ras2* and *tubulin*). In RIP ChIP experiments, antibodies against Sgf11 immunoprecipitated mRNA even more efficiently than did antibodies against Xmas-2 or ENY2 components of the mRNA-binding AMEX complex. In accordance with these results, we found Sgf11 to be essential for *hsp70* mRNA and general mRNA export. The effect of Sgf11 RNAi knockdown on general mRNA export was prominent, although weaker than that of Xmas-2 knockdown.

We have also found that Sgf11 interacts with the AMEX mRNA export complex and, similar to it, co-localizes with NPC. Along with AMEX, Sgf11 is also required for the export of most polyA mRNAs. Therefore, Sgf11–AMEX interactions may play a significant role in general mRNA export.

To elucidate the mechanism of Sgf11 functioning, we made an attempt to purify Sgf11-containing complexes from the embryonic nuclear extract and succeeded in identifying a complex in which Sgf11 was associated with the Cbp80 subunit of CBC. This result was confirmed in co-immunoprecipitation experiments. Moreover our data on co-expression of recombinant Sgf11 and Cbp80 in *Drosophila* cell culture demonstrated that Sgf11 directly interacts with Cbp80; this interaction was independent on the Cbp20 subunit of CBC. In line with the Cbp80–Sgf11 interaction, both proteins were associated with the *hsp70* promoter in the RNA-dependent manner. Moreover Cbp80 was necessary for Sgf11 recruitment.

**Figure 7.**
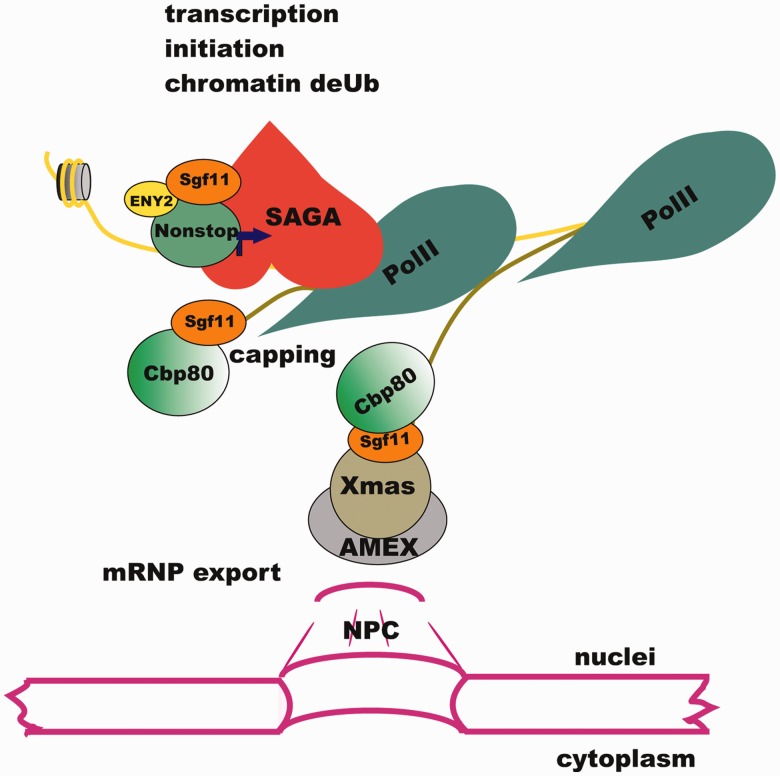
Sgf11 participates in different steps of gene expression in the nucleus. Sgf11 is localized on the promoter of the *hsp70* gene as a component of DUBm of SAGA. Following transcription activation, Sgf11 interacts with the Cbp80 component of the CBC and associates with the growing transcript in proximity to chromatin. Sgf11 associates with mRNP, interacts with the AMEX mRNA export complex associated with NPC and is essential for mRNA nuclear export.

Our model suggests that AMEX interacts with Cbp80-bound Sgf11 at the 5′ end of mRNA, and this interaction is essential for efficient mRNP export (Figure 7). According to data of other authors, Cbp20 binds to the cap structure at the 5′ end of nascent mRNA, whereas Cbp80 is involved in the interaction with cap-associated proteins, in particular, those participating in mRNA export (37,39–42). For instance, THO, a component of the mRNA transcription elongation and export complex (TREX), associates with Cbp80 via the Aly component of TREX (38,43). It may well be that Cbp80 interacts with different mRNP formation and export machines, such as TREX or AMEX, at different steps of mRNP biogenesis.

The available data suggest that Sgf11, similar to Cbp80 or AMEX, may be a component of mRNP particles. It has been shown that CBC remains in the mRNA particle until being replaced by eIF4E/G in the cytoplasm (44–46). Both Sgf11 and AMEX in approximately equal amounts form a dot-like pattern both in the nucleus and in the cytoplasm (16) (Figure 4A). A plausible scenario is that Sgf11 remains to be associated with mRNP in the cytoplasm, where it is present in a significant amount. The function of Sgf11 in the cytoplasm is under investigation.

Our data demonstrate that RNAi knockdown of Nonstop component of DUBm had no effect on mRNA export from the nuclei. However, DUBm is likely to have an indirect function in mRNA export. It has been shown that yeast DUBm interacts with the TREX-2 mRNA export complex, a homolog of *Drosophila* AMEX (47). The loss of yeast Sgf73 partially disrupts interactions between Sus1 and TREX-2 that leads to mislocalization of Sus1 to the cytoplasm and disturbs mRNA export (14,47).

Thus, the DUBm appears to link SAGA-dependent transcription with the nuclear export of mRNA in yeasts.

Sgf11 functions independently of DUBm in mRNA export. In fact, the other subunits of DUBm were shown to function independently of DUBm as a whole. Thus, Sus1/ENY2 was shown to present in several different complexes (16,18). Human ataxin 7, the homolog of the yeast Sgf73 subunit of DUBm, was found in cytoplasm and is involved in the regulation of cytoskeletal dynamics (48).

Taken together, our data provide evidence that although *Drosophila* Sgf11 is an integral component of SAGA DUBm, it also forms a complex with Cbp80 and associates with nascent mRNA. It interacts with the AMEX mRNA export complex and is involved in competent mRNP translocation to the cytoplasm.

## SUPPLEMENTARY DATA

Supplementary Data are available at NAR Online: Supplementary Figures 1–3.

## FUNDING

Molecular and Cellular Biology Program of the Russian Academy of Sciences; Russian Foundation for Basic Research [project nos 10-04-01820, 11-04-91339]; Scientific School Support Program [project no NSh-2814.2012.4]; a fellowship from the Centre for Medical Studies, University of Oslo, Russia (to A.N.K. and Y.V.S.); Federal Program “Scientific cadres of innovative Russia” [proposal 2012-1.1-12-000-1001-066]; Experiments were performed using the equipment of IGB RAS facilities supported by the Ministry of Science and Education of the Russian Federation [16.552.11.7067]. Funding for open access charge: Molecular and Cellular Biology Program of the Russian Academy of Sciences.

*Conflict of interest statement*. None declared.

## Supplementary Material

Supplementary Data
