# Supplementary material for: The DUBm subunit Sgf11 is required for mRNA export and interacts with Cbp80 in Drosophila
Source: Nucleic Acids Res. 2012 Sep 18;40(21):10689–700. doi: 10.1093/nar/gks857 (PMC3510517; doi:10.1093/nar/gks857)
Supplement: Supplementary Data [file supp_40_21_10689__index.html]

The DUBm subunit Sgf11 is required for mRNA export and interacts with Cbp80 in Drosophila — The DUBm subunit Sgf11 is required for mRNA export and interacts with Cbp80 in Drosophila — Supplementary Data 

# The DUBm subunit Sgf11 is required for mRNA export and interacts with Cbp80 in *Drosophila*

## Supplementary Data

files

**Files in this Data Supplement:**

- Supplementary Data - pdf file
