# Supplementary material for: The DUBm subunit Sgf11 is required for mRNA export and interacts with Cbp80 in Drosophila
Source: Nucleic Acids Res. 2012 Sep 18;40(21):10689–700. doi: 10.1093/nar/gks857 (PMC3510517; doi:10.1093/nar/gks857)

SUPPLEMENTARY MATERIAL

Supplementary Figure 1.

(A) Antibodies against Sgf11 raised in two rabbits recognize the same bands in nuclear extract from Drosophila embryos.

(B) Nonstop binds to multiple sites on polytene chromosomes from Drosophila salivary glands. Chromosomes stained with anti-Nonstop antibodies and co-stained with DAPI, and merged images are shown.

(C) The presence of Nonstop on the hsp70 promoter region was analyzed by ChIP assay of chromatin from normal cells (NT) and from cells after heat shock (HS) or after heat shock with RNase treatment (HS+RNase). The results of ChIP are shown as a percentage of input. Light gray shading indicates the background (baseline) level determined as the average of measurements in three noncoding sequences (see Materials and Methods). Nonstop occupancy of the hsp70 promoter increased after heat shock (HS) and was insensitive to RNase treatment. The level of Nonstop detected in ChIP experiments was reproducible but fairly low. The effect was the same when different anti-Nonstop antibodies or different ChIP protocols were used. A probable explanation is that Nonstop was masked by other proteins.

Supplementary Figure 2.

(A) The influence of the Sgf11 RNAi knockdown on hsp70 transcription in cells exposed to heat shock. The levels of Sgf11 and hsp70 transcripts were measured by quantitative RT-PCR in cells treated with GFP dsRNA (control) or Sgf11 dsRNA. The level of each transcript in control cells was taken as unity.

(B) The bigger field of the Sgf11-depleted and control cells from the experiment shown in Fig. 3F.

(C) The influence of Sgf11 RNAi knockdown with dsRNA corresponding to full-length Sgf11 mRNA (full) or its different fragments (1_1 and 1_2, see Materials and Methods) on the polyA RNA nuclear export (red). Nuclei were stained with DAPI (shown as green). Control cells were treated with GFP dsRNA.

(D) The efficiency of Sgf11 RNAi knockdown with dsRNA corresponding to full-length Sgf11 mRNA (full) or its different fragments (1_1 and 1_2) (see supplementary Fig. 2C) as estimated at the RNA and protein levels by quantitative RT-PCR and Western-blot analysis, respectively.

Supplementary Figure 3.

(A) Specificity of polyclonal antibodies against Cbp80 raised in rabbits as tested by Western blot analysis of nuclear extract from Drosophila embryos. Lanes stained with (PI) preimmune sera, (Im) immune sera, and (Ab) affinity purified polyclonal antibodies are shown.

(B) The influence of Cbp80 RNAi knockdown on the hsp70 transcription level in cells exposed to heat shock. The levels of Cbp80 and hsp70 transcripts were measured by quantitative RT-PCR in cells treated with GFP dsRNA (control) or Cbp80 dsRNA. The level of each transcript in control cells was taken as unity.

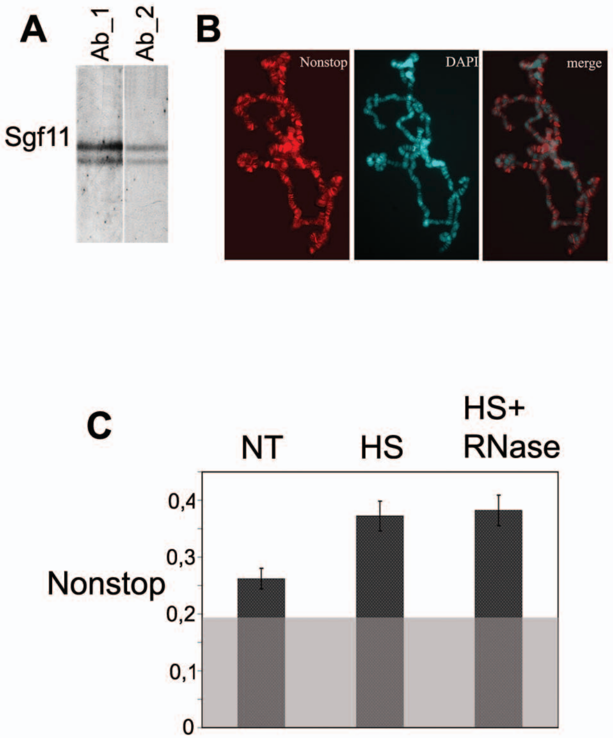
A
Ab_1
Ab_2
Sgf11
B
Nonstop
DAPI
merge
C
NT
HS
HS+
RNase
Nonstop
0,4
0,3
0,2
0,1
0

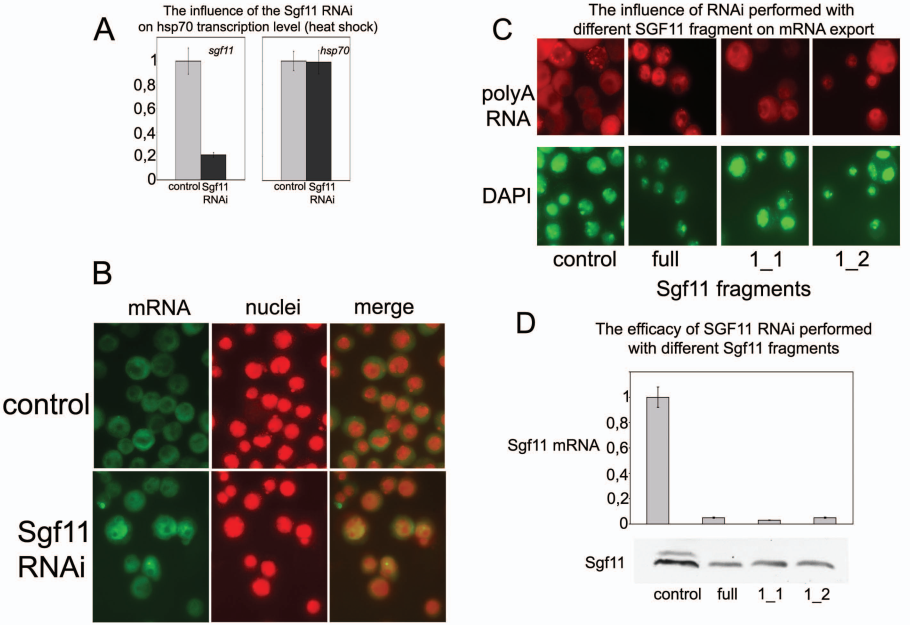
A
The influence of the Sgf11 RNAi
on hsp70 transcription level (heat shock)
sgf11
hsp70
1
0,8
0,6
0,4
0,2
0
control
Sgf11 RNAi
control
Sgf11 RNAi
B
mRNA
nuclei
merge
control
Sgf11 RNAi
C
The influence of RNAi performed with
different SGF11 fragment on mRNA export
polyA RNA
DAPI
control
full
1_1
1_2
Sgf11 fragments
D
The efficacy of SGF11 RNAi performed
with different Sgf11 fragments
Sgf11 mRNA
1
0,8
0,6
0,4
0,2
0
Sgf11
control
full
1_1
1_2

A

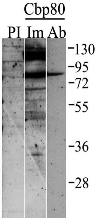

B

The influence of Cbp80 RNAi knockdown
on the hsp70 transcription level (heat shock).

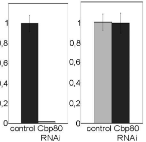

Supplement: Supplementary Data [file supp_gks857_nar-00988-a-2012-File009.pdf]
